# Assessment of symptoms of the post-COVID-19 syndrome in patients with different rheumatic diseases

**DOI:** 10.1186/s43166-023-00190-0

**Published:** 2023-04-27

**Authors:** Zahraa Nour Eldine Ismail, Mohamed Ahmed Hefny, Ahmed Elarabi Hendi, Marwa Gamal Tawfik

**Affiliations:** 1 Department of Physical Medicine, Rheumatology, and Rehabilitation, Faculty of Medicine, Suez Canal University, Ring Road, Ismailia, 41522 Egypt; 2 Department of Psychiatry and Neurology, Faculty of Medicine, Suez Canal University, Ismailia, 41522 Egypt

**Keywords:** Rheumatic disease patients, Post-COVID-19 syndrome, COVID-19

## Abstract

**Background:**

Patients with rheumatic diseases significantly suffer during and after infection with coronavirus disease (COVID-19). Post-COVID-19 syndrome (PCS) refers to signs and symptoms occurring during or following a COVID-19 infection that continue beyond 12 weeks. The study aimed to assess PCS symptoms in rheumatic disease patients compared to a control group not suffering from a rheumatic disease or any other chronic illness.

**Results:**

The prevalence of PCS symptoms was significantly higher in rheumatic disease patients compared to the control group: fatigue (69.1% vs. 41.25%), myalgia (73.5% vs. 37.5%), attention deficits (57.4% vs. 40%), and muscle weakness (33.8% vs. 13.8%). Objectively, the study group had significantly higher scores for the Fatigue Severity Scale (FSS) (35.46 ± 13.146 vs. 25.1 ± 7.587), Short-form McGill Pain Questionnaire (SF-MPQ-2) (21.66 ± 10.3 vs. 11.6 ± 3.433), and higher grades of functional disability in the Post-COVID-19 Functional Status scale (PCFS). Rheumatic disease patients had significantly higher frequencies of anxiety and depression, as assessed by the Hospital Anxiety and Depression Scale (HADS), and cognitive impairment, as assessed by the Mini-Mental State Examination (MMSE), than the controls (*P* = 0.023, *P* = 0.003, *P* = 0.0001, respectively). Moreover, SLE patients had the most symptoms and the highest FSS, SF-MPQ-2, PCFS, and HADS scores, as well as the lowest MMSE scores (*P* = 0.0001 for all except cough (*P* = 0.043), weakness (*P* = 0.015), paresthesia (*P* = 0.027), and anosmia (*P* = 0.039)). Lower disease duration, hospitalization during acute COVID-19, steroid use, smoking, and biologics non-use were significantly associated with higher PCS symptoms. Smoking was a significant risk factor (*P* = 0.048), and biologics use was protective (*P* = 0.03). Rheumatic disease patients who received two doses of the COVID-19 vaccinations had better scores on the FSS, HADS for anxiety and depression, and MMSE than those who received a single dose (*P* = 0.005, *P* = 0.001, *P* = 0.009, *P* = 0.01).

**Conclusion:**

Rheumatic disease patients have a higher prevalence and risk of PCS, so strict follow-up, avoiding smoking, controlling disease activity, and COVID-19 vaccinations are essential for decreasing the morbidity of PCS.

## Background

Coronavirus disease 2019 (COVID-19), caused by severe acute respiratory syndrome coronavirus 2 (SARS-CoV-2), began in the province of Wuhan in early December 2019. It was declared a pandemic by the World Health Organization (WHO) on March 11, 2020 [1]. Patients with an acute COVID-19 infection experience a wide range of symptoms, from minor respiratory symptoms to severe pneumonia necessitating mechanical ventilation and progressing to acute respiratory distress syndrome or multi-organ failure [2].

Unfortunately, although most COVID-19 infections are recoverable, many patients still experience ongoing COVID-19 symptoms after infection or even develop new symptoms. Recently, studies have revealed a high incidence of persistent symptoms after acute infection, resulting in the terms "long COVID" or "post-COVID-19 syndrome (PCS)" [2].

According to the National Institute for Health and Care Excellence (NICE), the Scottish Intercollegiate Guidelines Network (SIGN), and the Royal College of General Practitioners (RCGP), PCS refers to "signs and symptoms occurring during or following a COVID-19 infection that continue for beyond 12 weeks" [3]. Greenhalgh et al. classified PCS as a COVID-19-related condition that lasts longer than three weeks after symptoms begin [4].

The most frequently reported PCS symptoms are anxiety, depression, abnormal breathing, abdominal symptoms, chest/throat pain, fatigue, headache, cognitive problems, and myalgia [5]. Various symptoms have been reported within the PCS, demanding long-term follow-up [6].

Conflicting results were found regarding the risk and severity of COVID-19 infection in patients with rheumatic diseases; some studies reported that those patients have greater COVID-19 severity and are more liable to complications [7] due to the immunopathogenesis of COVID-19, while others did not note that [8]. Therefore, our purpose was to assess PCS symptoms in a sample of patients with different rheumatic diseases compared to a control group without rheumatic diseases or other chronic diseases.

## Methods

### Patients and study design

The study was a case–control study and included sixty-eight adult rheumatic disease patients and eighty age- and gender-matched individuals without rheumatic diseases or other medical comorbidities as a control group. Both groups had a history of COVID-19 infection during the preceding 3–8 months, confirmed by polymerase chain reaction (PCR) testing. The study was conducted in the Rheumatology Clinic of Suez Canal University from June to October 2022. Rheumatic disease patients met the classification criteria of their disease, including rheumatoid arthritis (RA) [9], systemic lupus erythematosus (SLE) [10], psoriatic arthritis (PsA) [11], axial spondyloarthritis (axSpA) [12], systemic sclerosis (SSc) [13], and Sjögren's syndrome (SjS) [14]. Subjects with other comorbidities, such as diabetes, renal illness, liver disease, thyroid problems, major psychiatric or mental disorders, and malignancies, were ruled out. The study was approved by the Suez-Canal University Ethical Committee Board (5040#) in accordance with relevant principles of the Declaration of Helsinki (2000 revision). Patients provided their written informed consent to participate in this work.

All subjects underwent complete history taking, including an inquiry about smoking, chronic illnesses, current medications, COVID-19 vaccinations (doses, types, and timing in relation to infection), and hospitalization during acute COVID-19. Rheumatic disease patients were assessed for disease activity: disease activity score using C-reactive protein (CRP) (DAS28-CRP) [15] in RA patients, SLE disease activity index (SLEDAI) [16] in SLE patients, disease activity in psoriatic arthritis (DAPSA) [17] in PsA patients, and ankylosing spondylitis disease activity score (ASDAS) [18] in axSpA patients.

The PCS symptoms were assessed subjectively in both groups. Each participant was asked about current fatigue, cough, dyspnea, anosmia, attention defects, muscle weakness, myalgia, and headache. In addition, PCS symptoms were assessed objectively in both groups using valid questionnaires. Participants were asked to fill out the following:

- Arabic-validated Fatigue Severity Scale (FSS)

It is a self-reported, valid questionnaire consisting of nine statements that rate the severity of fatigue in terms of how it affects motivation, exercise, and physical function. Each statement has a score between 1 and 7. Higher scores indicate more severe fatigue; a total score of 36 or more means fatigue [19].

- Arabic-validated Short Form-McGill Pain Questionnaire-2 (SF-MPQ-2)

The SF-MPQ-2 is the revised version of the SF-MPQ after including relevant neuropathic symptoms and changing the rating scale from 0 to 3 to 0 to 10. It assesses the severity of pain during the last week on a 23-item scale. The total score is the sum of all scores; the higher the score, the greater the severity of the pain [20].

- Arabic version of the Post-COVID-19 Functional Status Scale (PCFS)

The questionnaire assesses functional limitations during and after COVID-19 with the changes in lifestyle, sports, and social activities during the last week. The questions are scored from 0 to 4. A score of 0 means no functional limitations. Score 1 means a negligible change in all activities. Score 2 means that some activities can be independently achieved at a lower intensity. Score 3 means inability to complete specific tasks. Score 4 means unembellished functional restrictions. The overall disability grade is the highest reported score [21].

- Arabic-validated Hospital Anxiety and Depression Scale (HADS)

A valid self-reported questionnaire was initially designed to screen for anxiety and depression in non-psychiatric settings. It has two subscales: HADS-Anxiety and HADS-Depression. Each subscale consists of seven items with a four-point ordinal response. The total score of each subscale ranges from 0 to 21. The scores of 0–7 were normal; scores of 8–10 were borderline abnormal; and scores of 11–21 were abnormal [22].

- Arabic-validated Mini-Mental State Examination (MMSE)

It is a valid screening tool for cognitive impairment at evaluation time. It assesses orientation, registration, attention, recall, language, and constructional praxis. The total score is 30. Scores of 24–30 mean no cognitive impairment; scores of 18–23 mean mild cognitive impairment; and scores of 0–17 mean severe cognitive impairment [23].

Statistical analysis: It was done using the statistical package for the social sciences (SPSS) version 25. Results were presented as the mean ± SD or as a number (percentage). For data analysis, the following tests were used: Chi-square, Mann–Whitney U, Kruskal Wallis, post hoc, and regression analysis (binary logistic). The P was considered significant if ≤ 0.05.

## Results

Our study included a group of 68 rheumatic disease patients with a mean age of 43.19 ± 8.051 years, including 40 females and 28 males (F: M 1.42:1), and a control group of 80 age- and sex-matched subjects. The patients included 21 with RA, 17 with SLE, 11 with axSpA, 9 with PsA, and 10 with overlap syndrome: 4 SjS with overlapping features of RA, 5 SSc cases with features of SLE, and 1 with SLE and RA (rhupus).

In the study group, seven (10.29%) patients stopped their DMARDs during acute COVID-19 infection. Forty-one (60.29%) were vaccinated against COVID-19; 25 (60.9%) received two doses (all received the first dose at least four months before the infection and the second dose after the COVID-19 infection), and the others received one only (all received it post-acute infection and 2–6 months before the assessment). The received vaccines were Pfizer-BioNTech, Sinopharm, and Sinovac vaccines. The demographic and clinical characteristics of both groups are described in Table 1.

**Table 1:** Clinical and demographic characteristics of both groups

Parameter mean ± SD or n (%)	Case group (*n* = 68)	Control group (*n* = 80)	*P*
Age (years)	43.19 ± 8.051	43.26 ± 7.67	0.797^1^
Females: males	40:28 (1.42:1)	43:37(1.16:1)	0.423^2^
Disease duration (years)	5.1 ± 2.382	-	^**−**^
Smoking	31(45.6)	30 (37)	0.061^2^
Steroids use	50 (73.52)	-	**-**
Biologics use	16 (23.5)	-	-
Stop DMARDs (acute infection)	7 (10.29)		
Hospitalization: Yes	24 (35.29)	12 (15)	**0.002**^**2**^
No	44 (64.7)	68 (45.9)	
COVID-19 vaccinated	41 (60.29)	68 (85)	**0.001**
COVID-19 vaccination doses			
Single dose	16 (39.1)	19 (27.94)	0.23
Two doses	25 (60.9)	49 (72.06)	
Post-COVID-19 symptoms:			
Cough	22 (32.4)	13 (16.3)	**0.022**^**2**^
Dyspnea	30 (44.1)	22 (27.5)	**0.035**^**2**^
Weakness	23 (33.8)	11 (13.8)	**0.004**^**2**^
Fatigue	47 (69.1)	33 (41.25)	**0.001**^**2**^
Attention problems	39 (57.4)	32 (40)	**0.035**^**2**^
Myalgia	50 (73.5)	30 (37.5)	**0.0001**^**2**^
Headache	15 (22.1)	9 (11.3)	0.075^2^
Paresthesia	23 (33.8)	16 (20)	**0.037**^**2**^
Anosmia	6 (8.8)	3 (3.8)	0.198^2^
Post-COVID-19 syndrome			
1–3 symptoms	27 (41.2)	59 (81.9)	**0.0001**^**2**^
≥ 4 symptoms	38 (58.5)	13 (18.1)	
No symptoms	3 (4.41)	8 (10)	
FSS score	35.46 ± 13.14	25.1 ± 7.587	**0.0001**^**1**^
Fatigue (in FSS)	31 (45.6)	19 (23.8)	**0.005**^**2**^
SF-MPQ-2	21.66 ± 10.32	11.6 ± 3.433	**0.03**^**1**^
PCFS: grade 1	16 (23.5)	47 (58.8)	**0.0001**^**2**^
Grade 2	17 (25)	17 (21.3)	
Grade 3	23 (33.8)	9 (11.3)	
Grade 4	12 (17.6)	7 (8.8)	
HADS (Anxiety)	12.49 ± 3.509	9.99 ± 2.297	**0.0001**^**1**^
Normal	8 (11.8)	16 (20)	**0.023**^**2**^
Borderline	20 (29.4)	35 (43.8)	
Abnormal	40 (58.8)	29 (36.3)	
HADS (Depression)	12.46 ± 3.418	10.39 ± 2.410	**0.0001**^**1**^
Normal	8 (11.8)	12 (15)	**0.003**^**2**^
Borderline	20 (29.4)	43 (53.8)	
Abnormal	40 (58.8)	25 (31.3)	
MMSE score	21.44 ± 3.284	24.26 ± 1.447	**0.0001**^**1**^
Normal	20 (29.4)	63 (78.8)	**0.0001**^**2**^
Mild impairment	36 (52.9)	17 (21.3)	
Severe impairment	12 (17.6)	0	

^1^ = Mann–Whitney U test, ^2^ = Pearson's chi-squared test. *COVID-19* coronavirus disease 2019, *FSS* fatigue severity scale, *HADS* Hospital Anxiety and Depression Scale, *SF-MPQ-2* Short-form McGill Pain Questionnaire, *PCFS* Post-COVID-19 Functional Status scale, *MMSE* Mini–Mental State Examination, bold values are significant at *P* ≤ 0.05

The frequency of PCS symptoms, except headache and anosmia, was higher in the patient group than in the control group. Patients had significantly higher FSS, SF-MPQ-2, PCFS, HADS (depression and anxiety), and lower MMSE scores (cognitive impairment).

**Fig. 1:**
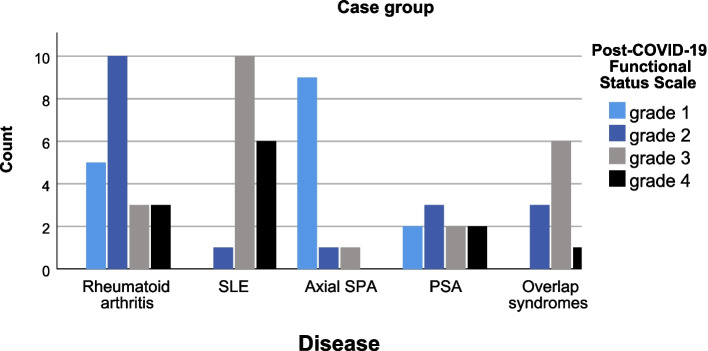
Systemic lupus erythematosus (SLE) patients showed higher grades of functional disability in post-coronavirus disease 2019 (COVID-19) functional status scale (PCFS) than other rheumatic diseases

Regarding the rheumatic disease type, significant differences were noticed in all PCS symptoms (except dyspnea) and all objective assessments; SLE patients and overlap syndromes patients reported the highest frequency of symptoms. SLE patients recorded the highest FSS, SF-MPQ-2, PCFS, and HADS scores and the lowest MMSE scores. In contrast, the axSPA group had the lowest scores (Table 2 and Fig. 1).

**Table 2:** Comparison of post-COVID-19 syndrome symptoms in rheumatic diseases patients

Parameter mean ± SD or n (%)	RA *n* = 21	SLE *n* = 17	axSpA *n* = 11	PsA *n* = 9	Overlap *n* = 10	*P*
Cough	2 (9.5)	9 (52.9)	5 (45.4)	2 (22.2)	4 (40)	**0.04**^**1**^
Dyspnea	11 (52.3)	11 (64.7)	2 (18.18)	2 (22.2)	4 (40)	0.08^1^
Weakness	6 (28.5)	10 (58.8)	0 (0)	2 (22.2)	5 (50)	**0.015**^**1**^
Fatigue	13 (61.9)	16 (94.1)	2 (18.2)	8 (88.8)	8 (80)	**0.0001**^**1**^
Attention problem	11 (52.3)	17 (100)	1 (9.1)	4 (44.4)	6 (60)	**0.0001**^**1**^
Myalgia	17 (80.9)	17 (100)	1 (9.1)	7 (77.7)	8 (80)	**0.0001**^**1**^
Headache	1 (4.7)	10 (58.8)	0 (0)	0 (0)	4 (40)	**0.0001**^**1**^
Paraesthesia	9 (42.8)	8 (47.05)	0 (0)	1 (11.1)	5 (50)	**0.027**^**1**^
Anosmia	0 (0)	4 (23.5)	0 (0)	0 (0)	2 (20)	**0.039**^**1**^
Autoantibodies						
ANA-IIF	4 (20)	15 (88.2)	0 (0)	2 (22.2)	9 (9)	
RF	15 (71.4)	2 (11.7)	0 (0)	(0)	4 (4)	
Anti-CCP	17 (81)	(0)	0 (0)	3 (33.3)	2 (2)	
Anti-dsDNA	0 (0)	13 (76.47)	0 (0)	0 (0)	2 (2)	
FSS score	32.1 ± 9.6	48.4 ± 9.7	19.5 ± 5.5	35.8 ± 10.1	37.9 ± 11.2	**0.0001**^**2**^
SF-MPQ-2	19.1 ± 9.4	30.5 ± 4.8	9 ± 2.9	20.4 ± 9.2	27.1 ± 8.8	**0.0001**^2^
HADS (anxiety)	12.1 ± 3	15.2 ± 2.5	8.1 ± 1.4	12.4 ± 4.2	13.4 ± 2.1	**0.0001**^**2**^
HADS(depression)	12.1 ± 2.6	15.2 ± 2.5	8 ± 1.4	12.2 ± 2.8	13.7 ± 2.8	**0.0001**^**2**^
MMSE score	22.1 ± 3.2	17.9 ± 1.4	25.3 ± 1.6	22.3 ± 1.7	21 ± 2.4	**0.0001**^**2**^
Disease activity	DAS28-CRP 4.1 ± 0.7	SLEDAI 12.7 ± 6.8	ASDAS 1.7 ± 0.4	DAPSA 23.6 ± 7		**-**

^1^ = Pearson's chi-square test, ^2^ = Kruskal–Wallis test. *RA* rheumatoid arthritis, *SLE* systemic lupus erythematosus, *axSpA* axial spondyloarthritis, *PsA* psoriatic arthritis, *FSS* fatigue severity scale, *HADS* hospital anxiety and depression scale, *SF-MPQ-2* short-form McGill pain questionnaire, *MMSE* mini–mental state examination, *DAS28* disease activity score, *SLEDAI* SLE disease activity index, *ASDAS* ankylosing spondylitis disease activity index, *DAPSA* disease activity of PsA. Bold values are significant at *P* ≤ 0.05

Rheumatic disease patients who had 1–3 PCS symptoms were compared to those with four symptoms or more in Table 3.

**Table 3:** Distribution of variables according to the number of post-COVID-19 symptoms in rheumatic disease patients

Parameter mean ± SD or n (%)	Post-COVID-19 symptoms		
	1–3 symptoms n (%) *n* = 27	≥ 4 symptoms n (%) *n* = 38	*P*
Gender:			
Males	10 (37)	15 (39.5)	0.8^1^
Females	17 (63)	23 (60.5)	
Age (years)	43.3 ± 6.5	43.6 ± 9.04	0.74^2^
Disease duration (years)	8.4 ± 4.2	6.6 ± 4.2	**0.02**^**2**^
Hospitalization	4 (14.8)	18 (47.4)	**0.006**^**1**^
Biologics use	12 (44.4)	1 (2.6)	**0.0001**^1^
DMARDs stopping	2 (7.4)	6 (10.5)	0.66^1^
Steroids use	14 (51.9)	36 (94.7)	**0.0001**^**1**^
Steroids dose:			
Low (< 7.5 mg/d)	6 (42.9)	10 (62.5)	0.32^1^
Medium (7.5–30 mg/d)	8 (57.1)	22 (61.1)	
High (> 30 mg/d)	0 (0)	4 (11.1)	
COVID-19 vaccinated	18 (66.7)	23 (60.5)	0.613^1^
Smokers	8 (29.6)	23 (60.5)	**0.014**^1^

^1 ^Pearson's chi-squared test. ^2^ Kruskal–Wallis test. *COVID-19* coronavirus disease 2019. Bold values are significant at *P* ≤ 0.05

**Fig. 2:**
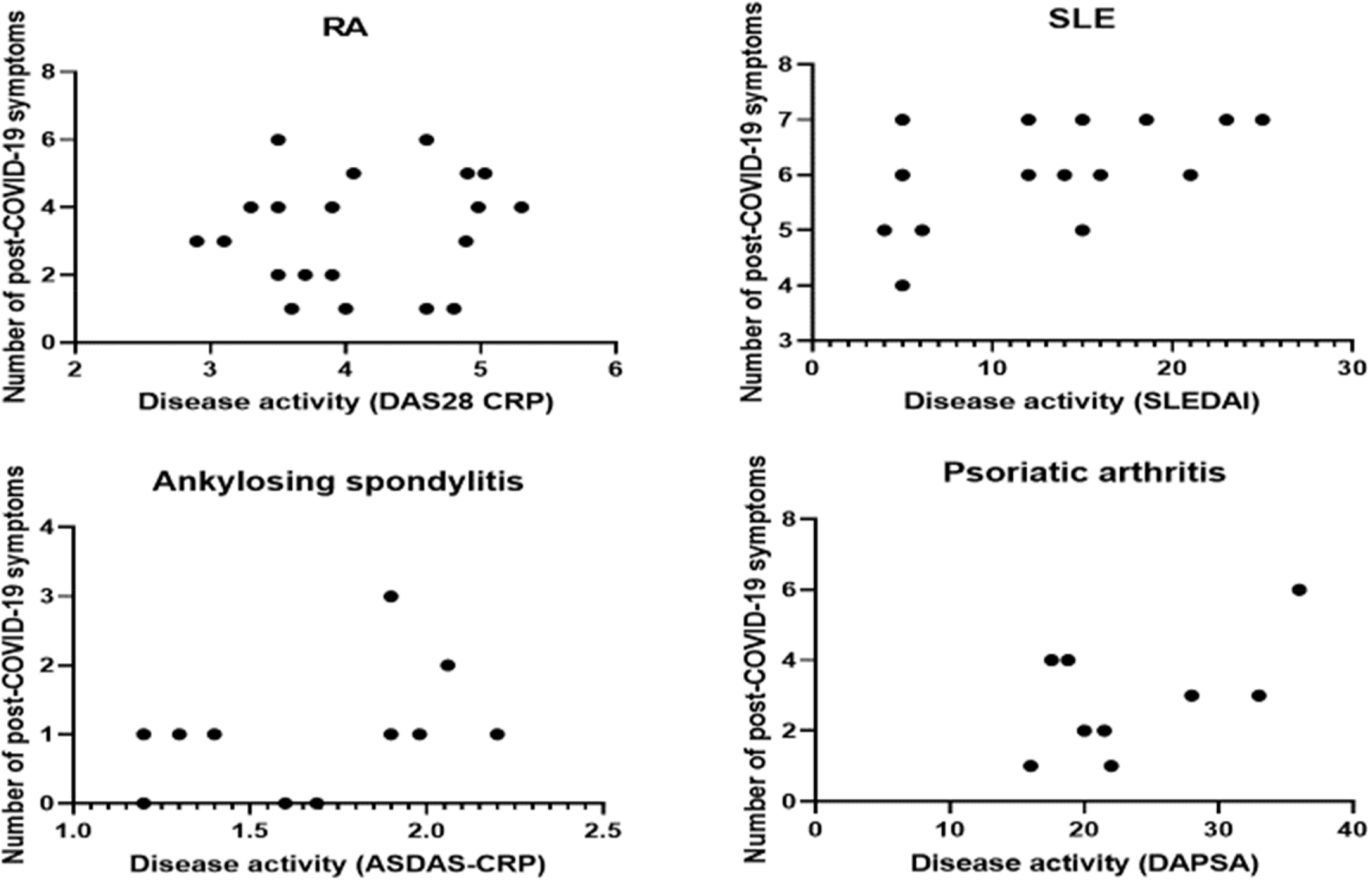
Non-significant correlations between the number of post-COVID-19 symptoms and disease activity as measured by the disease activity score (DAS28-CRP) in RA, the SLE disease activity index (SLEDAI), the disease activity in psoriatic arthritis (DAPSA), and the ankylosing spondylitis disease activity score (ASDAS) (*r* *=* 0.181 with *P* = 0.432, *r* = 0.433 with *P* = 0.083, *r* = 0.458 with *P* = 0.156, and* r = 0.3 with P *= 0.425, respectively)

Moreover, no significant correlations were noticed between the number of PCS symptoms and the disease activity calculated in RA, SLE, axSpA, and PsA patients (Fig. 2). In a binary logistic regression analysis model, smoking was a significant risk factor for higher PCS symptoms, while biologics use was a protective factor (Table 4). The association between COVID-19 vaccination doses and the severity of PCS symptoms in rheumatic disease patients was assessed in Table 5.

**Table 4:** Logistic regression analysis for predictors of ≥ 4 post-COVID-19 syndrome symptoms in rheumatic disease patients

Predictors	B	95% CI		*P* value
Disease duration	-0.105	0.756	1.073	0.241
Steroids	1.033	0.044	2.871	0.154
Hospitalization	1.111	0.065	1.658	0.178
Smoking	1.389	0.063	0.99	**0.048**
Biologics use	-2.834	1.276	226.627	**0.032**
Constant	0.417			0.775

Bold values are significant at *P* ≤ 0.05

**Table 5:** Post-COVID-19 syndrome severity in rheumatic disease patients according to vaccination doses received

Parameter mean ± SD	Single vaccine dose	Two vaccine doses	*P* value
FSS score	42.56 ± 10.91	32.48 ± 11.192	**0.005**
SF-MPQ-2	26.25 ± 9.09	19.64 ± 10.037	0.091
HADS (anxiety)	14.56 ± 2.828	11.28 ± 2.836	**0.001**
HADS(depression)	14.31 ± 3.049	11.44 ± 3.31	**0.009**
MMSE score	19.75 ± 3.1	22.4 ± 2.843	**0.012**

The used test was Kruskal–Wallis test. *FSS* fatigue severity scale, *HADS* hospital anxiety and depression scale, *SF-MPQ-2* short-form McGill pain questionnaire, *MMSE* mini–mental state examination. Bold values are significant at *P* ≤ 0.05

## Discussion

Post-COVID syndrome greatly burdens patients, causing significant disability and low quality of life. To our knowledge, few studies have tackled persistent COVID-19 symptoms in patients with rheumatic diseases. So, this study aimed to assess PCS symptoms in this group compared to control subjects not suffering from rheumatic diseases.

The current study refers to a higher frequency of PCS symptoms in rheumatic disease patients than in the control group. In harmony with our results, a Turkish study reported the PCS symptoms in 36 (67.9%) rheumatic disease patients; 41.5% had three or more symptoms, while 26.4% had one or two symptoms [24]. This study differs from the current study; it was a cross-sectional study that assessed the PCS symptoms subjectively only, without objective assessments for PCS symptoms.

These findings support the hypothesis that SARS-CoV-2 persistence in the body after infection resolution may induce some level of immune activation [25]. Another study reported long-lasting functional alterations of T cells as a possible cause of PCS [26]. Another article attributed the PCS to disrupted p38 mitogen-activated protein kinase (MAPK) signaling pathways that regulate cytokine production, causing dysfunctional peripheral and central cytokine inflammatory responses and autoimmunity [27]. So, autoimmune disease patients can be at high risk for PCS.

The emergence of autoimmune disease after COVID-19 [28] supports the immunological etiology of PCS.

In the present study, the most frequently reported symptoms in the patient group were myalgia and fatigue, compared to fatigue and attention problems in the control group. Batýbay et al. reported fatigue and weakness as the most frequent PCS symptoms in rheumatic disease patients [24]. A systematic review that included 40 studies of PCS in subjects without rheumatic diseases also identified arthralgia as the most common persistent symptom (65% of cases), followed by back pain (55%), and arm or leg heaviness (47%) [29]. This difference can be caused by the subjective assessment of symptoms.

Gamal et al. studied PCS in Egyptian subjects and reported that 38.82% had PCS symptoms. Post-viral fatigue was the most common symptom (23.5%), followed by arthralgia and myalgia (18.8%). The authors concluded that there were significant associations between PCS and age, the infection's severity, and chronic diseases [30].

Regarding the high frequency of anxiety, depression, and cognitive impairment in rheumatic disease patients, neuroinflammation [31], added to the already existing psychological burden of chronic autoimmune disease, may have contributed to such results.

Regarding the lower rates and severity of PCS symptoms in axSPA patients, the biological treatment of the assessed patients can be an explanation. These treatments may alleviate PCS symptoms by suppressing inflammatory cytokine dysregulation.

In our study, it was noticeable that some patients (26 patients, 38.23%) developed increased disease activity after COVID-19 infection. In addition, all the patients with high disease activity had severe symptoms. The explanation is that disease activity and its immune dysregulation can induce PCS. Furthermore, these symptoms can be caused by rheumatic disease activity. The insignificant correlations between the number of PCS symptoms and disease activity scores refer to the presence of other mechanisms and theories for PCS.

In this study, rheumatic disease activity-related characteristics, including steroid usage and low illness duration, were substantially associated with higher PCS symptoms, whereas biologics use was associated with fewer symptoms. Other factors, such as hospitalization and smoking, were associated with higher PCS symptoms. Whitaker et al. also reported a significant relationship between hospitalization and smoking, with a high frequency of persistent symptoms [32].

Regarding the insignificant association between the number of PCS symptoms and the COVID-19 vaccination in rheumatic disease patients, the assessment timing post-single dose was not sufficient for the long-term effect of the vaccine. Some of the patients were assessed two months after a single-dose vaccination.

The vaccination status in our study was low, and there was some dropout between doses 1 and 2 in rheumatic disease patients. Another study (in non-rheumatic disease patients) reported that two doses of BNT162b2 vaccination significantly correlated with a lower probability of PCS symptoms [33]. Watanabe et al. also reported the association between two-dose vaccination and a lower risk of PCS compared to no vaccination [34].

In the current study, the symptom severity in patients who received two doses of COVID-19 vaccines was significantly lower than in those who received only one dose. The majority (20 patients, 80%) reported improvement in PCS symptom severity after receiving the second dose of the COVID-19 vaccine. Nehme et al. reported a significant improvement post-vaccination in PCS sufferers (not rheumatic disease patients) [35]. Watanabe et al. reported that 20.3% of the subjects experienced symptomatic improvement after two weeks to six months of COVID-19 vaccination [34].

The improvement post-vaccination can be explained by the clearance of the remaining SARS-CoV-2 virus in the human body (viral remnant hypothesis of PCS) or the reduction of the dysfunctional immune response [36].

Good follow-up of rheumatic disease patients, controlling disease severity, smoking avoidance, and COVID-19 vaccinations are highly recommended to prevent severe, persistent sequelae of PCS.

This study had points of strength, as it was one of the few studies investigating PCS in rheumatic disease patients; the assessments were made subjectively and objectively using multiple assessment questionnaires. Furthermore, it assessed the association of COVID-19 vaccines with PCS in rheumatic disease patients, which is of great interest. The study also has limitations. The assessments used were all based solely on the patients' estimates. In addition, some PCS symptoms, such as fatigue, are highly prevalent in rheumatic disease patients; about 41 to 57 percent have fatigue [37].

## Conclusions

Rheumatic disease patients have a significantly higher frequency and risk of PCS symptoms. SLE patients and those with overlap syndromes reported the highest frequencies of symptoms. Smoking was a significant risk factor for higher PCS symptoms in rheumatic disease patients, and the biologics were protective. Moreover, patients who received two doses of COVID-19 vaccinations had lower PCS severity than those who received a single dose. So, good follow-up, controlling rheumatic disease severity, avoiding smoking, and receiving booster doses of COVID-19 vaccinations are essential to lowering PCS morbidity.

## Data Availability

The datasets analyzed during the current study are available with the corresponding author upon request.

## Abbreviations

COVID-19: Coronavirus disease 2019
PCS: Post-COVID-19 syndrome
SARS-CoV-2: Coronavirus 2
WHO: World Health Organization
NICE: National Institute for Health and Care Excellence
SIGN: Scottish Intercollegiate Guidelines Network
RCGP: Royal College of General Practitioners
RA: Rheumatoid arthritis
SLE: Systemic lupus erythematosus
PSA: Psoriatic arthritis
axSPA: Axial spondyloarthropathy
DAS28-CRP: Disease activity score using C-reactive protein
SLEDAI: Systemic lupus erythematosus disease activity index
DAPSA: Disease activity in psoriatic arthritis
ASDAS-CRP: Ankylosing Spondylitis Disease Activity Score with CRP
FSS: Fatigue Severity Scale
SF-MPQ-2: Short-form McGill Pain Questionnaire
PCFS: Post-COVID-19 Functional Status scale
HADS-A and HADS-D: Hospital Anxiety and Depression Scale
MMSE: Mini-Mental State Examination
MC: Mast cells
MCAS: Mast cell activation syndrome
MAPK: p38 mitogen-activated protein kinase
